# A review on the diagnosis of animal trypanosomoses

**DOI:** 10.1186/s13071-022-05190-1

**Published:** 2022-02-19

**Authors:** Marc Desquesnes, Marisa Gonzatti, Alireza Sazmand, Sophie Thévenon, Géraldine Bossard, Alain Boulangé, Geoffrey Gimonneau, Philippe Truc, Stéphane Herder, Sophie Ravel, Denis Sereno, Vincent Jamonneau, Sathaporn Jittapalapong, Philippe Jacquiet, Philippe Solano, David Berthier

**Affiliations:** 1 CIRAD, UMR INTERTRYP, 31076 Toulouse, France; 2 INTERTRYP, CIRAD, IRD, University of Montpellier, Montpellier, France; 3 National Veterinary School of Toulouse (ENVT), 23 Chemin des Capelles, 31000 Toulouse, France; 4 Department of Cell Biology, Simón Bolívar University, Caracas, 1080 Venezuela; 5 Department of Pathobiology, Faculty of Veterinary Science, Bu-Ali Sina University, 6517658978 Hamedan, Iran; 6 Zoonotic Diseases Research Center, School of Public Health, Shahid Sadoughi University of Medical Sciences, 8915173160 Yazd, Iran; 7 CIRAD, UMR INTERTRYP, 34398 Montpellier, France; 8 CIRAD, UMR INTERTRYP, Bouaké, Ivory Coast; 9 Pierre Richet Institute, National Institute of Public Health, BP1500, Bouaké, Ivory Coast; 10 CIRAD, UMR INTERTRYP, Dakar, Senegal; 11 Senegalese Institute of Agricultural Research, National Laboratory of Livestock and Veterinary Research, BP 2057, Hann, Dakar, Senegal; 12 IRD INTERTRYP, CIRAD, University of Montpellier, Montpellier, France; 13 Faculty of Veterinary Technology, Kasetsart University, Bangkok, 10900 Thailand

**Keywords:** Antibody-detection, Cross-reactions, DNA detection, Microscope examination, Trypanosome, Undetected infection

## Abstract

This review focuses on the most reliable and up-to-date methods for diagnosing trypanosomoses, a group of diseases of wild and domestic mammals, caused by trypanosomes, parasitic zooflagellate protozoans mainly transmitted by insects. In Africa, the Americas and Asia, these diseases, which in some cases affect humans, result in significant illness in animals and cause major economic losses in livestock. A number of pathogens are described in this review, including several Salivarian trypanosomes, such as *Trypanosoma brucei* sspp. (among which are the agents of sleeping sickness, the human African trypanosomiasis [HAT]), *Trypanosoma congolense* and *Trypanosoma vivax* (causing “Nagana” or animal African trypanosomosis [AAT]), *Trypanosoma evansi* (“Surra”) and *Trypanosoma equiperdum* (“Dourine”), and *Trypanosoma cruzi*, a Stercorarian trypanosome, etiological agent of the American trypanosomiasis (Chagas disease). Diagnostic methods for detecting zoonotic trypanosomes causing Chagas disease and HAT in animals, as well as a diagnostic method for detecting animal trypanosomes in humans (the so-called “atypical human infections by animal trypanosomes” [a-HT]), including *T. evansi* and *Trypanosoma lewisi* (a rat parasite), are also reviewed. Our goal is to present an integrated view of the various diagnostic methods and techniques, including those for: (i) parasite detection; (ii) DNA detection; and (iii) antibody detection. The discussion covers various other factors that need to be considered, such as the sensitivity and specificity of the various diagnostic methods, critical cross-reactions that may be expected among Trypanosomatidae, additional complementary information, such as clinical observations and epizootiological context, scale of study and logistic and cost constraints. The suitability of examining multiple specimens and samples using several techniques is discussed, as well as risks to technicians, in the context of specific geographical regions and settings. This overview also addresses the challenge of diagnosing mixed infections with different *Trypanosoma* species and/or kinetoplastid parasites. Improving and strengthening procedures for diagnosing animal trypanosomoses throughout the world will result in a better control of infections and will significantly impact on “One Health,” by advancing and preserving animal, human and environmental health.

**Graphical Abstract:**

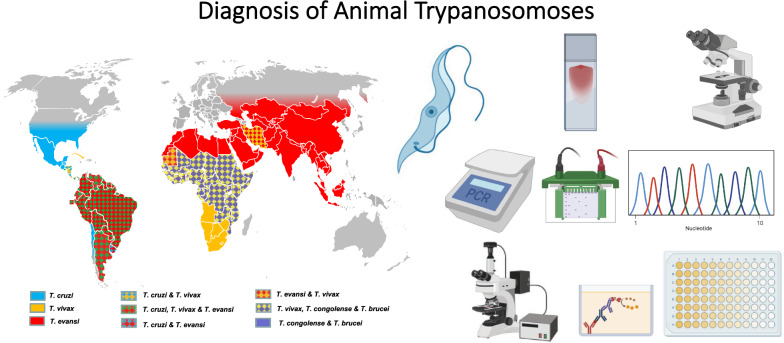

## Background

The family Trypanosomatidae (phylum Protozoa, class Kinetoplastida) comprises 14 monoxenous genera infecting insects (e.g. *Leptomonas*, *Herpetomonas*) and five dixenous genera having invertebrates as vectors (one genus infecting plants [*Phytomonas]* and the other four infecting animals and humans). Among the four genera infecting animals and humans, two are anecdotic, i.e. *Endotrypanum* found in sloths and *Porcisia* found in porcupine; the other two genera, namely *Trypanosoma* and *Leishmania*, contain pathogenic parasites of medical and veterinary importance. Both *Trypanosoma* and *Leishmania* are widely distributed over the world, and they affect humans, animals or both, as anthroponotic or zoonotic agents [1]. In humans, leishmaniasis has a high medical impact, with approximately 70,000 deaths recorded annually and an estimated 350 million individuals at risk [2]. In animals, leishmanioses seriously affect dogs, while in cattle and horses, the impact is limited [3, 4]. Trypanosomoses are significant diseases of domestic and wild mammals, affecting millions of livestock in Africa, the Americas and Asia [5], as well as of humans in Africa and Latin America [6–10].

Among the 125 *Trypanosoma* species found in mammals, 10% are considered to be pathogenic to humans and/or other mammals [11]. These pathogenic trypanosomes mainly inhabit the host’s blood and lymph, but do occur sometimes in the cerebrospinal fluid (CSF) and other host tissues, and some of them have intracellular stages. Trypanosomes are mainly transmitted by insects, but alternative means of transmission include mammals as vectors (e.g. vampire bats for *Trypanosoma evansi* and marsupials for *Trypanosoma cruzi*) and transcutaneous and transmembrane routes, such as peroral, venereal, intraplacental, iatrogenic, routes, among others, which allow occasional horizontal and vertical transmission.

The major pathogenic trypanosomes, approximately 10 species, subspecies or types, originate from Africa, where they are mainly cyclically transmitted by the bite of tsetse flies (as Salivarian trypanosomes) [11]; two subspecies are zoonotic, while the others are “animal parasites,” infecting wild and domestic mammals, including livestock, despite scarce occurrences in humans [12]. One other important pathogenic trypanosome of mammals occurs in the Americas, i.e. *T. cruzi* (subgenus* Schizotrypanum*) where it is responsible for a neglected tropical disease (NTD) named “Chagas disease” that extends into South and Central America. As a Stercorarian trypanosome, *T. cruzi* is biologically transmitted through the feces of triatomine bugs, and host contamination occurs by transmembrane or transcutaneous penetration. *Trypanosoma cruzi* is not only a human pathogen but also a zoonotic parasite, affecting a huge range of domestic and wild mammals, including livestock, as reported in a recent review [13]. In this review, we focus on the diagnosis of trypanosomes in animals; however, in a “One Health concept” [14], this focus includes human pathogens, providing that animals carry and/or are affected by these pathogens, and/or play a role in the epidemiology of the human diseases (reservoir or screen). We also cover the so-called “atypical human infections by animal trypanosomes” [12].

## Definitions and geographical distributions

### African trypanosomoses

Animal trypanosomoses of African origin (ATAO) are known under several disease names that are associated with one or several *Trypanosoma* species involved. “Nagana” is a disease complex caused by one or several Salivarian trypanosomes belonging to subgenera* Nannomonas* (*Trypanosoma congolense*, *T. simiae* and *T. godfreyi*)* Duttonella* (*Trypanosoma vivax* and *T. uniforme*; the occurrence of the latter needs to be confirmed) and* Trypanozoon* (*Trypanosoma brucei brucei, T. brucei gambiense* and *T. brucei rhodesiense*) “Surra” is a disease caused by *T. evansi*, and “Dourine” is caused by *Trypanosoma equiperdum* (sometimes referred as *T. brucei evansi* and *T. brucei equiperdum*, due to their phylogenetic relations) [11, 15–19].

**Fig. 1:**
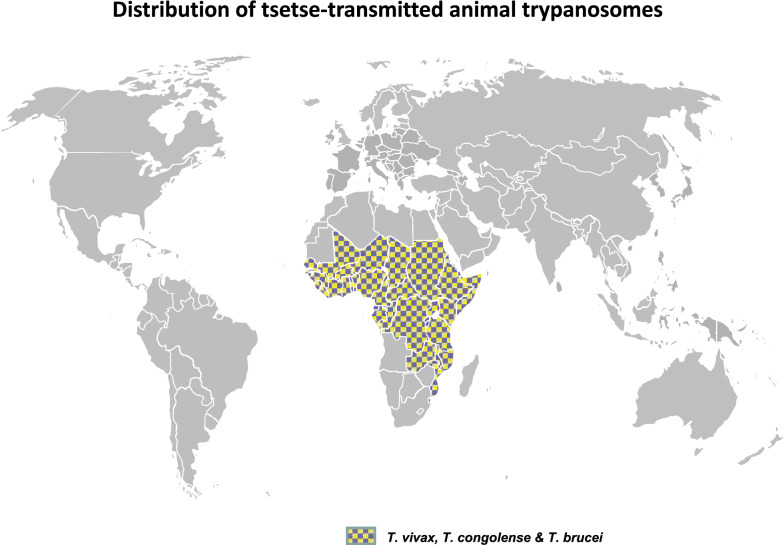
Geographical distribution of the “Nagana” disease complex (*Trypanosoma congolense*, *T. vivax* and *T. brucei*) [32–36]

Apart from the last two species that will be discussed below, *Trypanosoma* spp. responsible for Nagana are mainly cyclically transmitted by flies of the genus *Glossina* (tsetse flies). Their geographical distribution is determined by the geographical distribution of the tsetse fly, which is restricted to specific areas of Africa (Fig. 1). Tsetse flies have been reported to occur in an area estimated to be 10 million km^2^ in size, in 37 countries, in humid and sub-humid sub-Saharan part of Africa (from latitude 10° N to 20°–30° S and in some limited areas of the Arabian Peninsula [5, 20, 21]. Nagana affects both wild and domestic mammals, but the impact of infection is the highest in cattle, with about 3 million livestock dying annually despite the administration of approximately 35 million doses of trypanocidal drugs. The economic losses in cattle production is estimated to be about US$ 1.0–1.2 billion [22]. Although the most pathogenic agent of Nagana for livestock is *T. congolense* type Savannah [23], *T.* *vivax* is the most prevalent. To a lesser extent and with a somehow enigmatic impact, *T. brucei brucei* also contributes to the disease complex. Moreover, the human pathogens *T. b. gambiense* and *T. b. rhodesiense* have been found in livestock [24–29], but their pathogenic effects and impact are not yet thoroughly clarified. However, experimental infections of cattle with *T. b. rhodesiense* were found to lead to a fatal central nervous syndrome in half of the cases tested [30]. In addition to cattle, other species, such as goats, sheep, pigs and dogs, may be affected. Horses and camels are very susceptible to Nagana; indeed, in the past, tsetse flies used to constitute a natural barrier preventing the introduction of camels and horses into the southern Sahel regions of Africa [31].

**Fig. 2:**
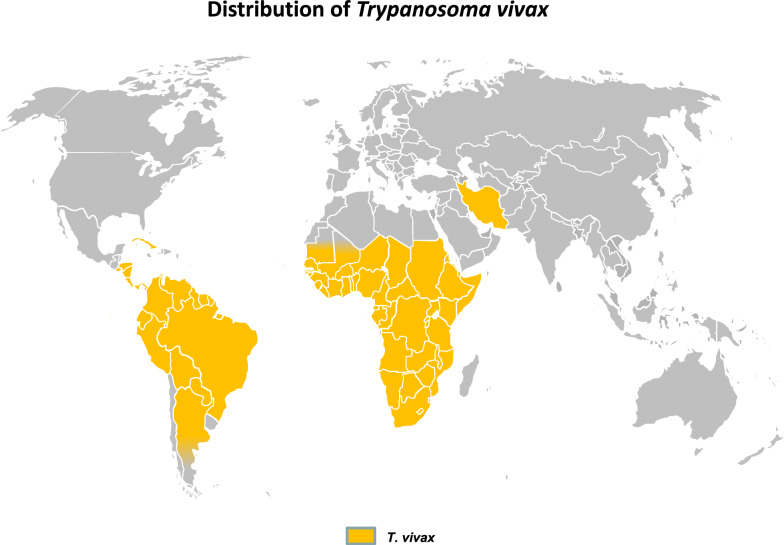
Geographical distribution of *Trypanosoma vivax* [6, 32–34, 36, 42]

Some of these *Trypanosoma* spp. may also be mechanically transmitted by biting flies, such as tabanids and Stomoxyine flies [37, 38], especially *T. vivax*, as first suspected [39] and later confirmed in semi-liberty conditions [40], and also *T. evansi*, which is discussed in subsequent sections. Mechanical transmission has allowed *T. vivax* to spread in some areas of Africa that are free of or were cleared of tsetse (e.g. in Ethiopia [41]). Similarly, during the eighteenth century, *T. vivax* invaded South and Central America [6], and more recently it was reported in Iran [42]. Geographical distribution of *T. vivax* is presented in Fig. 2.

**Fig. 3:**
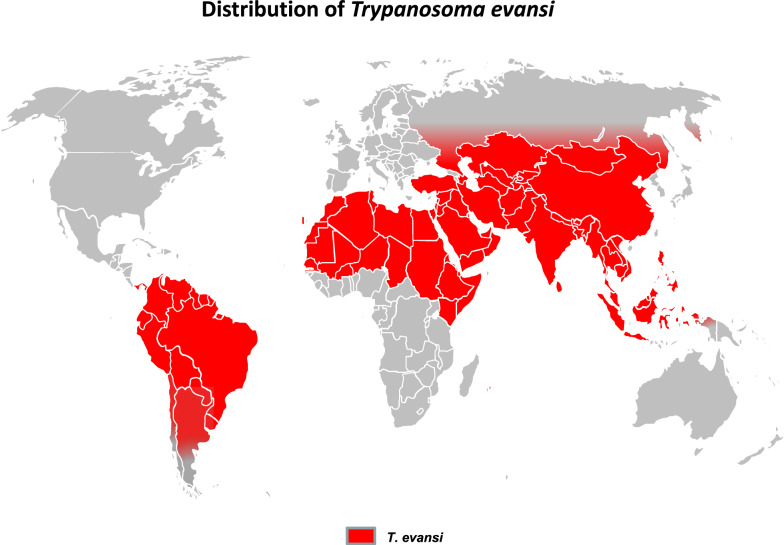
Geographical distribution of *Trypanosoma evansi* (“Surra”) [6, 33, 43, 50]

The other members of the ATAO are the two *Trypanozoon* derived from the *T. brucei* lineage, but these are not transmitted cyclically. *Trypanosoma evansi*, the causative agent of Surra, is mechanically transmitted by tabanids and Stomoxyine flies and found in tropical areas, but also ranges up to Mongolia. Surra significantly affects camels and horses in Africa and Latin America, but also cattle and buffaloes in Asia [43]. Surra is the most widely distributed animal trypanosomosis, ranging from South to Central America, the upper half of Africa, Middle East and Asia (Fig. 3). *Trypanosoma equiperdum* is the causative agent of Dourine, a venereal disease transmitted worldwide among equids [44]. In the last decade it was described in Italy [45], Mongolia [46], Ethiopia [47] and Iran [48], but its geographical distribution is mostly unknown. These two species may be found in the same hosts and in the same areas as the agents of Nagana, which interfers with species-specific diagnosis. Together with *T. vivax*, these trypanosomes are responsible for the “non-tsetse-transmitted animal trypanosomoses” (NTTAT) [49].

Clinical signs of ATAO may include intermittent fever, anemia, edema, abortion, decreased fertility, emaciation and death; genital and neurological symptoms are also possible. However, none of these symptoms are pathognomonic. Clinically, ATAO can be confused with other parasitic diseases (i.e. babesiosis, anaplasmosis, among others), rabies, plant intoxications and *T. cruzi* infection in Latin America. Therefore, case identification must rely on diagnostic techniques that: (i) confirm the presence of trypanosomes, with by microscopic visualization or by obtaining evidence of trypanosome DNA, or (ii) demonstrate a host—parasite contact through antibody detection techniques [51]. Differential diagnosis is based on observations and evolution of clinical signs, epidemiological context and, most critically, by laboratory test results.

Human African trypanosomiasis (HAT), or sleeping sickness, is caused by two *T. brucei* subspecies transmitted by tsetse flies, with a substantial socio-economic impact in humans, although it is still classified as a NTD [9, 52]. If not treated, sleeping sickness is usually a fatal disease. *Trypanosoma b. gambiense* is responsible for a primarily anthroponotic disease form that accounts for 98% of HAT cases [53, 54]. It results in a chronic disease that can last several years, and occurs in West and Central Africa. Some animals have been reported as potential reservoirs [26, 29], but their exact role in the epidemiology of the human disease is not clear [28, 55]. Based on molecular characterization, a variant of *T. b. gambiense* has been described recently, leading to the consideration of variants Tbg1 and Tbg2, and possibly more, within *T. b. gambiense* [56]. In East Africa, where the zoonotic *T. b. rhodesiense* is responsible for an acute disease form in humans, representing 2% of HAT cases [53, 54], the parasite is also found in a wide range of wild and domestic animals, including cattle, goats and pigs, which act as reservoirs [27, 57–59]. About 70 million people worldwide are at risk of sleeping sickness [53]. In addition to the socio-economic impact on exposed populations, the presence of these two subspecies creates a risk of infection for farmers, veterinarians and slaughter-house workers as well as laboratory technicians, when handling meat, carcasses and blood. Consequently, in these areas of Africa, animal samples should be handled with appropriate biosafety and containment procedures. The existence of these two forms of HAT also creates a need for subspecies-specific diagnosis methods in order to evaluate the human risk and to investigate, and possibly control, the animal reservoirs. Under the expanding “One Health” concept, identifying these zoonotic agents in livestock (and wild fauna) may prove useful to control HAT [60, 61].

### American trypanosomosis

**Fig. 4:**
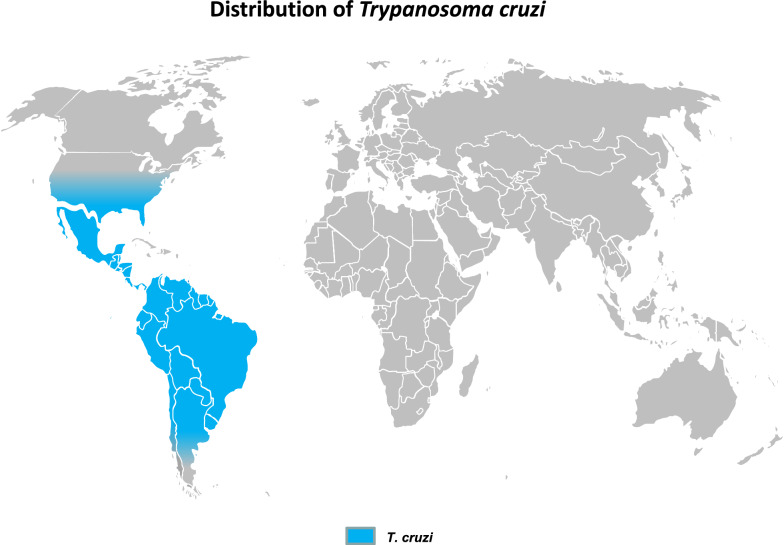
Geographical distribution of *Trypanosoma cruzi* [6, 69, 76]

*Trypanosoma cruzi*, the agent of the American human trypanosomiasis, or Chagas disease, is mainly transmitted via feces of triatomine bugs, affecting 6–8 million people, mainly in Latin America, most of whom are chronic carriers [62], while 65–100 million people are at risk [63]. Other ways of transmission are of variable importance, such as vertical (mother to fetus and mother to child) and iatrogenic transmission, especially through blood transfusion and organ transplantation [64]. Detecting the potential presence of *T. cruzi* in blood collected from Latin American people who lived previously in endemic areas is a serious concern, notably in Europe and the USA [10]. Acute human cases were reported recently to be linked to peroral infection via fruit juices contaminated by the bug’s feces. This route of infection, possibly under-detected in the past, now appears to be a significant mode of transmission [65–68]. Although *T. cruzi* is considered to be a human pathogen, it has a large wild animal reservoir that includes marsupials, armadillos, raccoons, squirrels, wild pigs, rats, among others, as well as a domestic reservoir, including dogs, cats, pigs, sheep, goats, cattle and horses [69]. The presence of *T. cruzi* may be a source of interference in animals being investigated for trypanosomoses, as already observed for *T. evansi*/*T. cruzi* infections in horses in Argentina [70]. Additionally, the possible presence of a human pathogen in animal samples constitutes a potential risk for human health at the farm and laboratory levels. *Trypanosoma cruzi* is a neglected but true pathogen in animals, and even though the production cycle of livestock is mostly too short to allow clear clinical expression of the disease (especially for short-cycle species such as pigs [71]) it may undergo a complete evolution and cause deleterious clinical signs in other animals; for example, myocarditis has been observed in dogs and nervous invasion with ataxia in horses [72–74]. The geographical distribution of Chagas disease in humans extends from mid-Argentina and Chile to Mexico; however, the geographical distribution of *T. cruzi* extends more northwards to include most of the southern USA and a broad area extending from northern California to northern Pennsylvania [75] (Fig. 4). Consequently, in these areas, special care should be taken when handling animal samples; laboratory work should be performed with appropriate biosafety and containment procedures, especially when horse, cattle, pig, dog or wild fauna samples are being handled as these may be infected with *T. cruzi* [69].

### Atypical human infections by animal trypanosomes

Very rare human cases caused by animal *Trypanosoma* species, including *T. vivax*, *T. congolense*, *T. b. brucei*, *T. evansi* and *T. lewisi* or *T. lewisi*-like, have been reported; these are referred to as as “atypical human infections by animal trypanosomes” (a-HT) [12, 77]. Among these, a growing number of human cases have been reported, particularly in Asia due to *T. evansi*, and *T. lewisi* (subgenus* Herpetosoma*). *Trypanosoma lewisi* is a cosmopolitan parasite of rats which has low pathogenicity in its original host [12]. Diagnostic methods for detecting *T. lewisi* in rats are thus needed for risk analysis and, conversely, diagnosis of this species may also be required in humans. Several human infections by *T. congolense *were recently exposed by PCR examination in a survey in Maro, southern Chad [78], suggesting that human infections with *T. congolense* might be more frequent than previously thought. In any case, these surprising results certainly need confirmation.

### Distribution of pathogenic trypanosomes

**Fig. 5:**
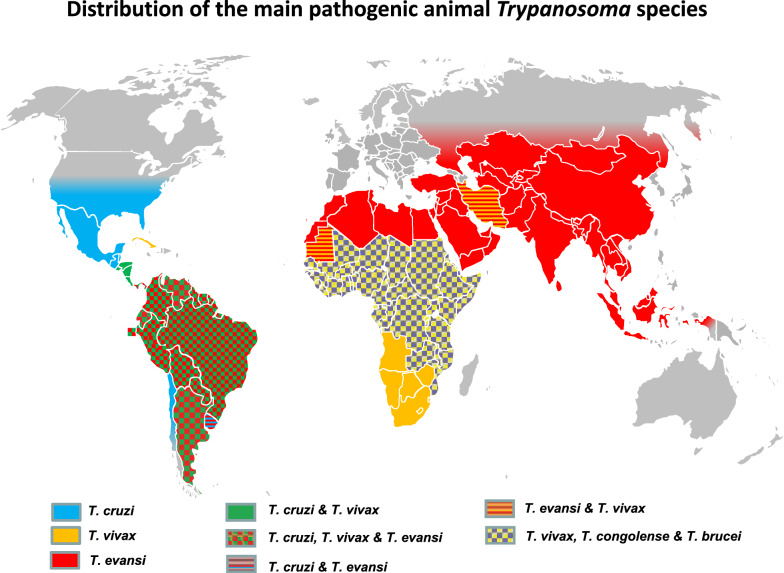
Geographical distribution of pathogenic mammal trypanosomes [6, 33–36, 42, 43, 50, 69, 76]

In addition to vector control, in the absence of protective vaccines or efficient prophylactic strategy, control of the diseases mentioned above strongly relies on detecting and treating positive cases. Diagnostic results not only support treatment decisions, but they are the basis for epidemiological studies, monitoring and evaluation of the efficiency of disease control measures. Furthermore, in the field of animal husbandry, diagnosis is also a tool used to implement health policy, including slaughter-hous policy, and to define the health status of animals prior to international movements. Finally, knowledge of those *Trypanosoma* species that are potentially present in the investigated area is necessary to consider interference in diagnosis and to prevent the risk of infection to humans handling these samples. To support such awareness, a tentative world distribution of pathogenic animal trypanosomes is represented in Fig. 5, mostly based on publications, including geographical reviews [6, 34–36, 43, 50].

## Review and characterization of available diagnosis techniques

Clinical signs of animal trypanosomoses are not sufficiently specific to support a clinical diagnosis and, therefore, laboratory tests are required to confirm clinical suspicions. As a consequence, case confirmations and epidemiological studies can only be carried out using laboratory facilities.

The course of a Salivarian trypanosome infection can vary from acute to chronic, but also be asymptomatic, depending on the intrinsic pathogenicity of the parasite and susceptibility, immune competence and health history of the host. One of the characteristics of pathogenic Salivarian trypanosome infections is a highly fluctuating parasitemia, which reflects the affinity of the parasite to tissues, and the control of the parasite population by the host immune system, which the parasite cyclically escapes by developing a population with new variable surface glycoprotein (VSG), the generation of which is referred to as “variable antigen type” (VAT). Consequently, when a VAT is recognized by the host immune system, the trypanosome population exhibiting this VAT is destroyed by the immune system and the parasitemia decreases drastically to undetectable levels for some time; when a new VAT population multiplies, parasitemia once again increases. This cycle is the reason why parasite concentrations in the host blood are highly variable from one day to another and sometimes even nil, or undetectable. As a general rule, parasitemia is high in early infections, lower and less frequent in chronic infections and nil or erratic in the case of subclinical evolution of the disease. Therefore, diagnosis efficacy can be seriously affected when performed at times of low parasitemia levels.

Numerous diagnostic tests are available to detect trypanosomes or diagnose trypanosomoses [79]. Current diagnostic tests vary in their sensitivity and specificity, the ease with which they can be implemented and their cost [80]. The choice of one or several particular tests is guided by epidemiologically-adapted diagnostic requirements, availability of equipment and expertise and economic principles. These choices will be discussed in another article in this journal  (“Proper use and perspective on diagnosis methods for animal trypanosomoses”). In the present review we describe the characteristics of the methods currently available for diagnosing animal trypanosomes. Apart from antigen detection, which despite numerous attempts remains unsuccessful, three types of diagnostic techniques for animal trypanosomes can be distinguished: (i) parasite detection; (ii) DNA detection; and (iii) antibody detection (Tables 1, 2). Technical information on these tests is available on the websites of the World Organization for Animal Health (OIE) [81] and of the Food and Agriculture Organization of the United Nations (FAO) [32]. A recently published “Compendium of standard diagnosis protocols for animal trypanosomoses of African origin” is available on the OIE website [13]. These documents can be found at the following links: https://www.oie.int/fileadmin/Home/eng/Health_standards/tahm/3.04.14_NAGANA.pdf; https://www.fao.org/3/X0413E/X0413E00.htm; https://www.oie.int/app/uploads/2021/06/compendiumstandarddiagnosticprotocolsanimaltrypansomosesafricanorigin-en.pdf , respectively.

**Table 1:** Summary of diagnostic techniques used for animal African trypanosomosis and non-tsetse transmitted animal trypanosomosis

Target	Technique^a^	Analytic sensitivity (parasite/ml)	Sensitivity (percentage estimation if available)	Specificity	Logistic needs: field/laboratory	Cost	Infection step	Scale	Livestock /fauna	Test objective and/or context
Parasite	Wet blood film	10^4^–10^5^ [80]	No data	+	Field: microscope	Low	Acute infection	Individual	Livestock (fresh blood)	Epidemiology or experimental follow-up
	HTC/BCM	2.5 × 10^2^–5 × 10^3^	+ (14–24) [82]	+ +	Field: microcentrifuge + microscope	Low	Acute sub-acute infection	Individual (group)	Livestock (fresh blood)	Individual diagnostic (epidemiology)
	mAECT		+	+ +	Field: microcentrifuge + microscope	High	Acute sub-acute infection	Individual	Livestock (fresh blood)	For research: parasite isolation
	Rodents inoculation	10–10^3^	+ + +	+ + +	Laboratory with animal facilities	High	Acute sub-acute infection	Individual	Livestock (fresh blood)	For research: parasite isolation
DNA	Pan-species primers (ITS1)	50 [83]	+ + (54–74) [84]	+ + may require sequencing (99–100) [84]	Laboratory for molecular biology	High	Acute sub-acute infection	Individual and group	Livestock and wildlife	Epidemiology
	Genus/species specific primers	1–10 [85]	+ + + (65–88) [84]	+ + + + (99–100) [84]	Laboratory for molecular biology	High	Acute sub-acute infection	Individual and group	Livestock and wildlife	Epidemiology
Antibodies	ELISA against total antigens		+ + + (90.5) [82]	+ +	Laboratory for serology	Low	Acute to chronic and past infection	Group (individual)	Livestock	Epidemiology (individual diagnostic)
	Protein specific ELISA		+ + + (98.9) [86]; (85.9–98.1) [87]	+ + + (98.9) [86]; (90.4–100) [87]	Laboratory for serology	Medium	Acute to chronic and past infection	Group (individual)	Livestock	Epidemiology (individual diagnostic)
	Agglutination test (CATT)		+ + (87) [88]; (44.5–95.2) [89]; (76–88) [90]; (32.4–52.9) [91]	+ + (81) [88]; (79.5–99.5) [89]; (92–98) [90]; (77.8–84.6) [91]	Field	Low	Acute to chronic and past infection	Individual (group)	Livestock	Individual diagnostic (epidemiology)
	IFAT		+ +	+	Laboratory for serology	Medium	Acute to chronic and past infection	Individual (group)	Livestock	Individual diagnostic
	CFT		+ + (31.5–73.5) [89]; (90–100) [92]	+ + (89.2–98.5) [89]; (89–99) [92]	Laboratory for serology	Medium	Acute to chronic and past infection	Group (individual)	Livestock	Individual diagnostic (epidemiology)
	TL		No data	+ +	Laboratory with animal facilities	Very high	Acute to chronic and past infection	Individual	Livestock	Individual diagnostic

Main test characteristics are indicated. Qualitative information is given for sensitivity and specificity, ranging from low (−) to high (+ + +) performance. Quantitative data are given when available. It should be noted that these values are not directly comparable since tests were performed on different samples sets, with different techniques and in different laboratories. Primary use is indicated first and additional use is given in parentheses

^a^See Abbreviation List for the full description of each abbreviation

**Table 2:** Summary of diagnostic uses for main *Tryanosoma* species responsible for animal African trypanosomosis and non-tsetse transmitted animal trypanosomosis

Target	Technique^a^	Subgenus* Nannomonas*	Subgenus* Duttonella*	Subgenus* Trypanozoon*		
		*Trypanosoma congolense*	*Trypanosoma vivax*	*Trypanosoma brucei brucei*	*Trypanosoma evansi*	*Trypanosoma equiperdum*
Parasite	Wet blood films	+^b^	+	+	+ *Trypanozoon*	
	HTC/BCT	+ + +	+ + +	+ + +	+ + + *Trypanozoon*	
	mAECT	−	−	−	−	−
	Inoculation of rodents	+	0	+	+ +	+
DNA	Pan-species primers (ITS1)	+ +	+ +	+ +	+ + *Trypanozoon*	+
	Genus/species/type-specific primers	+ + +	+ + +	+ + +	+ + + *Trypanozoon*	+ +
	Specific primers among *Trypanozoon*			0	+	0
Antibodies	ELISA on WCLSA	+ + +	+ + +	+ + +	+ + + *Trypanozoon*	+
	Protein-specific ELISA	Under test	Under test	0	+	0
	Agglutination test (CATT)	0	0	+	+ + +	0
	IFAT	−	−	−	−	+
	CFT	0	0	0	0	+ +
	TL	0	0	0	+	0

^a^See Abbreviation List for the full description of each abbreviation

^b^Qualitative uses: +  +  +  = well adapted and used; +  +  = adapted and used; +  = can be used; 0 = not used in practice

Finally, the four OIE reference laboratories on animal trypanosomoses may provide biological materials and technical training to support diagnostics (Table 3).

**Table 3:** World Organization for Animal Health reference laboratories for trypanosomes

Topics	OIE reference laboratories
Trypanosomoses (tsetse transmitted)/animal trypanosomes of African origin	CIRAD-Bios, UMR InterTryp (CIRAD-IRD), 34,398 Montpellier, cedex 5, France
Surra (*Trypanosoma evansi*)	National Research Center for Protozoan Diseases, Obihiro University of Agriculture and Veterinary Medicine, Obihiro, Japan
	Institute of Tropical Medicine Antwerp, B-2000 Antwerpen, Belgium
Dourine (*Trypanosoma equiperdum*)	ANSES, Laboratory for animal health, Normandy site, Unit Physiopathology & Epidemiology of Equine Diseases (PhEED), RD675, 14430 Goustranville, France

In this review, we primarily consider the methods recommended by the OIE (fully demonstrated and validated by large field applications), presenting their characteristics, performance, advantages, disadvantages and limitations.

### Parasite detection techniques

#### In the hosts

**Fig. 6:**
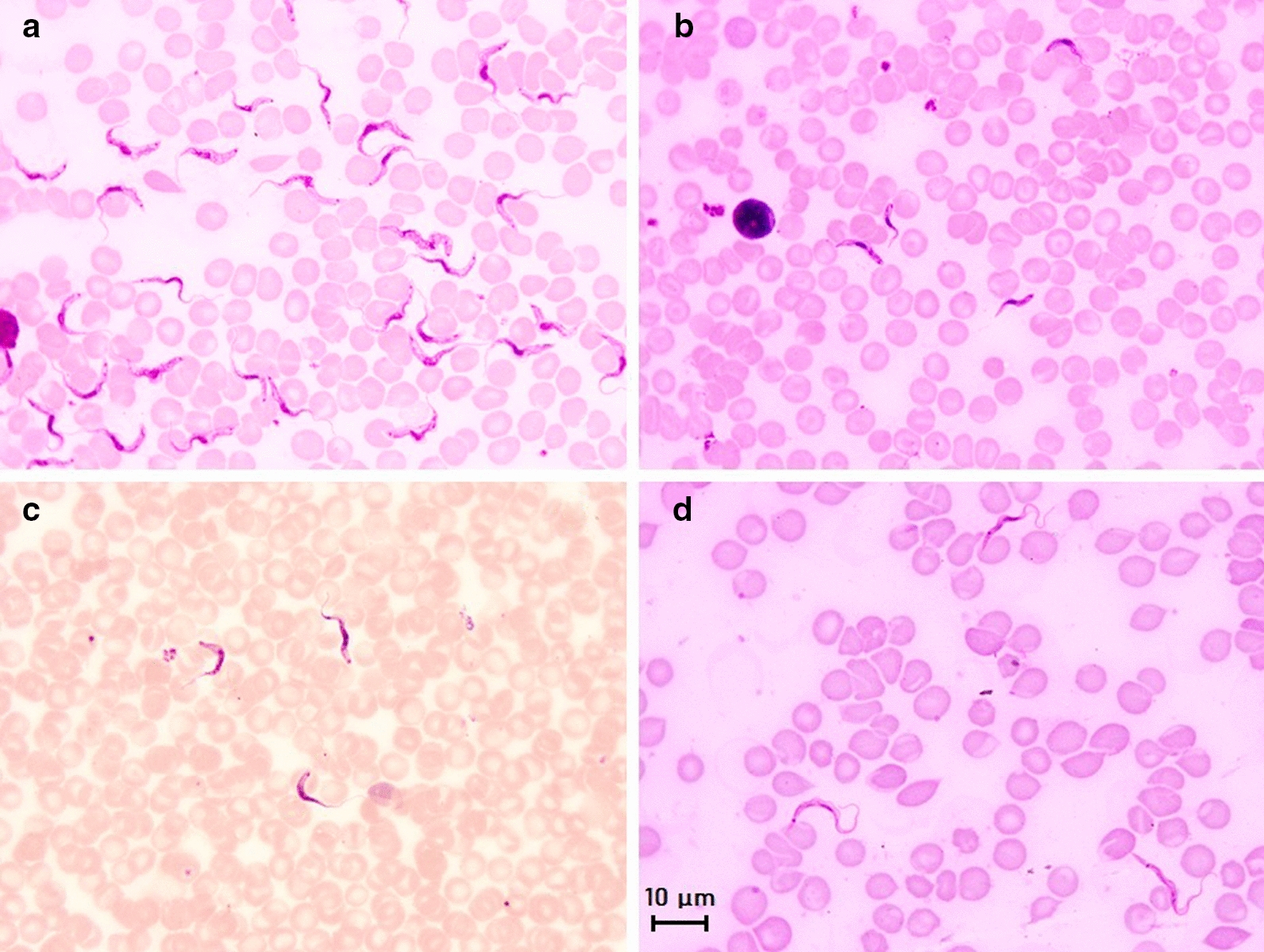
Main morphological features of four subgenera of mammal trypanosomes on Giemsa-stained thin blood smears. **a** Microscopic image of *Trypanosoma brucei brucei* in mice blood; morphology of the subgenus* Trypanozoon*: large-sized trypomastigote (17–30 µm), slender form, free flagellum, small sub-terminal kinetoplast, sharp posterior extremity, central nucleus and large undulating membrane; **b** Microscopic image of *Trypanosoma congolense*-type savanna in mice blood; morphology of the subgenus* Nannomonas*: small-sized trypomastigote (8–22 µm), no free flagellum, terminal sub-lateral kinetoplast, round posterior extremity, central nucleus and no undulating membrane. **c** Microscopic image of *Trypanosoma vivax* in cattle blood; morphology of the subgenus* Duttonella*: large-sized trypomastigote (20–27 µm), slender form, free flagellum, large terminal kinetoplast, round posterior extremity, central nucleus and large undulating membrane. **d** Microscopic image of* Trypanosoma lewisi* in rat blood; morphology of the subgenus* Herpetosoma*: very large-sized trypomastigote (21–36 µm), slender form, free flagellum, very large sub-terminal kinetoplast, very long sharp posterior extremity, anterior nucleus and large undulating membrane. Scale bar: 10 µm

Several direct parasite detection techniques based on microscopic examination can be used; ranked from the lowest to highest sensitivity these include: (i) microscopic examination of fresh wet blood films (simplest technique); (ii) the Giemsa-stained thin blood smear (GSBS), which allows identification to the subgenus level based on parasite morphology (Fig. 6); (iii) the hematocrit concentration technique (HCT), which uses a capillary tube, or the Woo method [93]; and (iv) the buffy coat method (BCM, Murray method) [94]. Thelatter is derived from the HCT, but the capillary tube is cut after centrifugation to extrude the buffy coat onto a microscope slide for examination. Concentration techniques exhibit a higher sensitivity (Table 1); however, the BCM has lower repeatability and reproducibility rates than the HCT due to varying levels of technician skills linked to the delicate extrusion and dropping of the buffy coat onto the slide that can in some instances vary in quality.

Parasite detection techniques require little equipment and are generally inexpensive, fast and easy to carry out although blood smear observation is time-consuming. Therefore, they are the techniques of choice to ascertain a *Trypanosoma* infection. The GSBS is subgenus specific, i.e. it is able to distinguish the subgenera* Nannomonas*,* Duttonella*,* Trypanozoon*,* Megatrypanum* (such as *Trypanosoma theileri*, a non-pathogenic trypanosome found in bovines and cyclically transmitted by tabanids as a Stercorarian parasite [11]),* Schizotrypanum* and* Herpetosoma* (Fig. 6). In addition, GSBS allows the study of parasite morphology and can faciliatedifferential diagnosis of other haemoparasitoses (e.g. *Babesia*, *Theileria*, *Anaplasma*). Both the HCT and BCM give immediate results, but HCT is better suited to screen large numbers of animals since it is the faster and more reproducible of the two tests. HCT and BCM also provide the packed cell volume (PCV), which estimates the level of anemia, one of the most critical indicators of trypanosomosis in cattle, among other hemoparasitoses, hemonchosis, etc. As trypanosomosis is a herd problem, the PCV profile can be used as a marker to orientate investigations. The PCV can also be used to decide whether a sample should be submitted for PCR examination, thereby limiting the total number of samples needed to be tested and increasing the probability of detecting infected animals. Nevertheless, the main drawbacks of these techniques are their very low analytic sensitivity, which depends on the concentration of the parasite in the sample and the volume of the sample examined (as low as 70 µl in capillary tubes and around 3–5 µl in direct blood examination and GSBS). Consequently, the sensitivity of parasitological techniques may vary from “very high” in early infection, when the animals are unable to control the parasitemia (91% for *T. evansi* in cattle [95, 96]), to “low” in chronic infections, when parasitemia is lower and transient (30–60% in sheep infected with *T. vivax*), and “nil” in healthy carrier situations when the animals can maintain the parasite at undetectable level in the blood [6] or in extravascular foci [97], including the skin [98]. BCM has been reported to have a very low individual sensitivity of around 14–24% [82]**.** Parasitological techniques are thus likely to miss chronic infections and asymptomatic carriers. At a population level, the sensitivity of parasite detection techniques is “high” during epizootic outbreaks, but “low” or “very low” in stable enzootic areas where most of the animals are in chronic or subclinical stages of the disease evolution. As an example of the latter: in French Guiana, despite regular sampling of about one-third of the cattle farms per year, *T. vivax* may not be observed during a 3- to 5-year period, before relapse occurs and the parasite being detected again on several farms [6].

There are other techniques based on parasite detection, and while these may be more sensitive, they are also more expensive and/or more time-consuming. In addition, they require specific skills and equipment that are not generally available. The use of anion exchange chromatography was thoroughly investigated and described in a study published in the 1970s [99]. It was recently reviewed and described again for the diagnosis of sleeping sickness [100]. The mini anion exchange centrifugation technique (mAECT), currently the most sensitive method to diagnose sleeping sickness [101, 102], can be applied to animal samples. However, the technique is cumbersome and is not suitable for the examination of a large number of samples. In vivo isolation of trypanosomes through intra-peritoneal injection of blood from a suspect animal to rodents, usually mice or rats, preferably immunosuppressed using cyclophosphamide [103], is expensive and time-consuming, diagnosis is not immediate and the method raises serious animal welfare concerns. Nevertheless, blood inoculation into rodents is more sensitive than HCT and some PCR procedures (e.g. tests targeting the internal transcribed spacer [ITS]); thus, blood inoculation is beneficial in revealing sub-patent infections and for parasite isolation [104]. However, the success rate of in vivo cultures depends on the *Trypanosoma* species involved: it is “highly sensitive” for the detection of* Trypanozoon* infections (especially *T. evansi*), of “medium sensitivity” for *T. congolense* strains, and generally “nil” or “scarcely effective” for *T. vivax*. On the other hand, the method is currently an essential tool for parasite isolation (parasite purification and enrichment before cryo-conservation) and the massive production of trypanosomes to prepare trypanosome antigens used in serological diagnosis or for subsequent molecular characterization. Procedures for in vitro cultivation of *Trypanosoma* spp. have also been described, but these require sophisticated equipment and protocols, the test results are not immediate (time delay) and they are certainly not suitable for large-scale studies. Competition among field stocks may also affect the results of in vitro cultures; for example, in the case of mixed infections, *T. theileri* easily overgrows *T. b. brucei* [105].

Overall, a negative result from parasitological examination does not unequivocally mean the absence of infection, as a negative result is not conclusive in terms of carrier/non-carrier since a *Trypanosoma* parasite may still be present and remain undetected. Such long periods of undetectable infection (1 year) despite a daily blood test (HTC) were observed in French Guiana, in a sheep experimentally infected with *T. vivax* and kept under a mosquito net [6].

Conversely, a positive parasitological result may verify the infection of an animal by a specific *Trypanosoma* parasite depending on the level of specificity allowed by the technique and the epizootiological context. For example, microscopic observation of a* Trypanozoon* on a GSBS from a cattle blood sample in Southeast Asia indicates *T. evansi*, while the same result in Africa can only indicate a* Trypanozoon* due to the potential presence of up to five species or subspecies of* Trypanozoon* on the African continent.

Consequently, detecting a single taxon cannot exclude the presence of a mixed infection, according to the geographical origin of the sample. Thus, in areas of possible mixed infections (Africa, Latin America), detection of an infection in an animal by a *Trypanosoma* will always leave open the possibility of mixed infection by one (or several) other *Trypanosoma* species or type, which may remain undetected, for several reasons (parasitemia below the detection threshold, extravascular refuge of the parasite, etc.). In conclusion, the status of an animal whose parasitological test results are positive should be considered as “animal infected by at least (the trypanosome subgenus, species or type detected), and possibly others”. Potential concomitant infection(s) will always lead to dubious situations in the field.

#### In the vectors

Microscopic observation of pathogenic trypanosomes may also be applied to cyclical vectors (tsetse flies and triatomine bugs); however, the morphology of trypanosomes in their cyclical vector is not characteristic, and the location of the parasite in the vector’s organs, such as gut, salivary glands or proboscis, may be confusing; consequently, such diagnoses have a very low species specificity. When molecular techniques were also applied, many parasitological diagnoses were shown to be wrong, probably due to a lack of sensitivity in the vector’s organ detection, but also due to the potential presence of many unidentified *Trypanosoma* spp. that may be found in the same vector’s organs, thus interfering with the diagnosis [106]. In addition, the dissection procedure is subject to a high risk of contamination, mainly between organs of the same vector, which could result in false positive organs by PCR due to the high sensitivity of the latter. Consequently, it is recommended that microscopic observation be used first to detect the trypanosomes in the vector’s organ, followed by molecular tools on these organs to identify the species or subspecies, similar to procedures used in hosts (see section In the hosts).

### DNA detection techniques

**Fig. 7:**
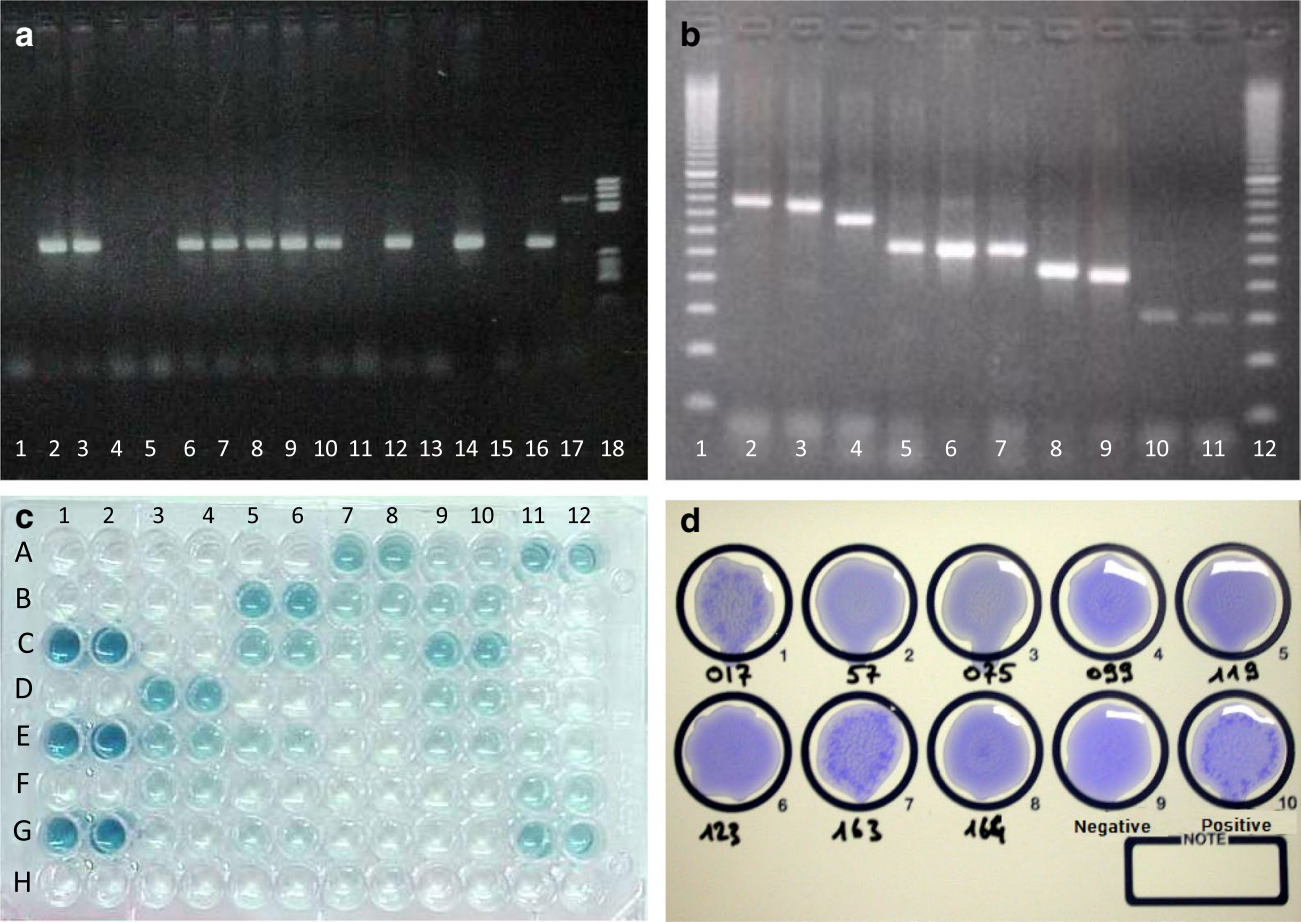
Molecular and serological tests for the detection of trypanosomes and trypanosomoses: **a** Ethidium bromide-stained electrophoresis gel of a monospecific PCR; the result can be considered to be positive when a visible PCR product exhibits the specific weight expected (here: lanes 2, 3, 6–10, 12, 14, 16); otherwise, when the product is non-specific (lane 17) or non-visible (lanes 1, 4, 5, 11, 13, 15), the PCR is negative. Lane 18 is the DNA ladder. **b** Ethidium bromide-stained electrophoresis gel of a multi-specific PCR based on the amplification of the internal transcribed spacer 1 (ITS1); species-specific results are deduced from the weight of the visible PCR products obtained (here: lanes 1, 12 are the DNA ladder; lanes 2–4 are T*. congolense*; lanes 5–7 are* Trypanozoon*; lane 8 is *T. theileri*; lane 9 is *T. simiae*; lanes 10, 11 are *T. vivax*. **c** *Trypanosoma vivax* antibody detection ELISA plate; first 2 rows are blanks (A, B), positive controls (C, E, G) and negative controls (D, F, H); all samples are tested in duplicate and appear to be positive, doubtful or negative, according to their mean optical density. **d** Picture of the card of a CATT/*T. evansi* exhibiting parasite agglutinations in the positive control and samples 163 and 017; other samples are considered to be negative

 DNA detection techniques used for trypanosomes can be applied to both the hosts and vectors. Several primer pairs have been designed for PCR amplification of trypanosome DNA. Based on highly repetitive satellite DNA (10,000–20,000 tandem repeats per genome), the gold standard primer sets available for the different trypanosome subgenera, species and types are, based to the OIE “Compendium of standard diagnosis protocols for animal trypanosomoses of African origin” [51]: TBR1 and TBR2 (subgenus* Trypanozoon*); TCS1 and TCS2 (*T. congolense* savannah type); TCF1 and TCF2 (*T. congolense* forest type); TCK1 and TCK2 (*T. congolense* Kenya Coast type [or Kilifi]); TSM1 and TSM2 (*T. simiae*); DGG1 and DGG2 (*T. godfreyi*) [107]; and TVW1 and TVW2 (*T. vivax*) [108, 109]. These monospecific PCRs are positive when the specific weight product expected is visible on the gel (Fig. 7a).

For *T. vivax*, several other primer sets have been published since the development of TVW primers [110–113], but none have proved to exhibit better sensitivity than the gold standard. In addition, it has been claimed by a few authors that some strains of *T. vivax*, notably from East Africa, may not be detected using TVW primers [110]; however, such parasites have never been isolated, and the results were never confirmed [114]. Consequently, to date, TVW primers remain the gold standard method for detecting and identifying *T. vivax*, but a well-designed comparison of the different published primers pairs on samples from different regions deserves to be performed.

Similarly, several primers have been developed to detect *T. evansi* [95, 115, 116], including nested and TaqMan primers [117, 118], but their sensitivity was found to be lower than that of TBR primers [85]. As per their specificity regarding other subspecies of the subgenus* Trypanozoon*, it was never fully documented. More interestingly, some primers were developed to distinguish Type A from Type B of *T. evansi* [83, 119]; these primers are helpful for obtaining accurate epidemiological information. More specific methods are also available to identify *T. b. gambiense* and *T. b. rhodesiense* [120–123], which could provide new information on the role of domestic and wild fauna in the maintenance of some sleeping sickness foci [26, 27, 29, 59, 124]. However, these single-gene targets are of low sensitivity [125]. Due to the diversity of taxon-specific primers in tsetse flies or mammalian hosts within the tsetse belt in Africa, a complete identification of *Trypanosoma* species may require three to six or even more PCR tests to be carried out per sample, which considerably increases the cost of the diagnosis.

In the Americas, and outside the tsetse belt in Africa, primers TBR and TVW are recommended for detecting *T. evansi* and *T. vivax*, respectively, for the diagnosis of trypanosomes in livestock. However, in horses, it is not possible tomake a definitive distinction between *T. equiperdum* and *T. evansi* using standard diagnostic tools. Clinical observations, analysis of the presence of vectors and information on the mode of transmission and overall epidemiological context are necessary to differentiate these two species. In fact, these parasites are so close that even molecular biology techniques barely differentiate them. At the present time, these two species tend to be considered as subspecies of *T. brucei*; *T. brucei evansi* and *T. brucei equiperdum* have polyphyletic origins, as shown by genomic studies [18, 19, 126]. In the Americas, TCZ1 and TCZ2 primers for detecting *T. cruzi* should also be used in addition to primers TBR and TVW [127]. Finally, a differential diagnosis for *Trypanosoma rangeli* may also be necessary in wild mammals [128].

Amplifications of the ITS1 of ribosomal DNA have been developed to allow the identification of all African *Trypanosoma* spp. in single or mixed infections using one single test [129–134], based on the specific weight of the PCR products obtained (Fig. 7b). These tests are helpful for screening; however, the sizing the PCR product(s) on gels can sometime be unreliable. Consequently, sequencing is most often required to confirm species identification, a procedure that is not suitable for routine diagnosis. Alternatively, ITS1 amplification can be used for sample screening and followed by monospecific PCR for species identification, when required. Finally, based on the highly conserved regions from which the primers have been designed, amplification of the ITS1 with TRYP1 primers allows the detection of all African pathogenic trypanosomes, as well as of *T. lewisi* [96, 134, 135] and even *T. cruzi* and *Leishmania* spp. (S. Ravel, personal communication) [136]. Loop-mediated isothermal amplification (LAMP) was also developed for trypanosome diagnosis [137]; however, the limited use of this technique does not fully validate veterinary usage.

PCR techniques require well-equipped laboratories and well-trained technicians. The quality of the results depends on the quality and quantity of DNA preparations and the choice of adequate primers, and the cost remains higher than parasitological techniques. However, depending on the context and the question to be answered, PCR techniques offer significant improvement in terms of sensitivity and specificity. The PCR is a highly sensitive method and typically provides a two- to threefold higher prevalence than parasitological methods when applied to field samples [6, 29, 138, 139]. In the best cases, analytical sensitivity of the PCR test for trypanosomes reaches as low as one to two parasites, or even less, per reaction [85, 139]. The sensitivity depends on the DNA targeted by the primers (repeated sequence or gene vs single gene) and the sample preparation method [140].

The specificity of PCR methods is theoretically very high, ranging from the subgenus to subspecies or type levels depending on the primer set used. PCR can also be used in vectors or in wildlife [141]. The gold standard PCRs are extremely sensitive. False-positive results may occur due to sample contamination with trypanosome DNA from real positive samples. False-negative results may occur when the parasitemia is very low (< 1–10 trypanosome/ml of blood), which is frequent in chronic infections and healthy carriers, when too much DNA was used in the PCR reaction or because of remaining inhibiting factors due to extraction of poor quality DNA. False-negative results may also be obtained when the specificity of the primers is too high, so that not all isolates of a particular *Trypanosoma* species are recognized. For example, in East Africa, the use of primers for the detection of the RoTat 1.2 VSG gene of *T. evansi* has undoubtedly left undetected the *T. evansi* Type B strains that are devoid of this gene [33, 142, 143].

LAMP techniques, sometimes promoted as “field methods,” are also very promising [144]; however, they remain in the hands of those laboratories that implemented their initial development and have never reached the stage of practical and widespread use, so they have never been really validated [143, 145, 146].

Real-time PCR (RT-PCR) methods have been developed for *T. evansi* [147], *T. brucei* [148] and *T. congolense* [149]; they were applied to hosts and vectors with high sensitivity and specificity, but their use has to date been very limited, probably due to associations with high technical skills and equipment and cost. These techniques have not yet been validated for routine diagnostic tests. Other attempts have been made for *T. b. gambiense* DNA detection, but the results of quantitative RT-PCRs were disappointing, with tests exhibiting a low sensitivity [150].

Spliced Leader trypanosome RNA (SL-RNA) detection was recently developed for the diagnosis of living *T. b. gambiense* in humans, but this technique has not been evaluated in animals so far [151].

As stated earliers in this review, the detection of pathogenic trypanosomes may be performed in the vectors using the same molecular techniques as used in hosts. However, sample preparation must be adapted to the insect organs of interest in order to get eliminate any PCR inhibitors present in insect samples, such as salivary glands, proboscis and midguts [109, 152] or fecal drop [153].

Finally, sample collection has been simplified by using blood or buffy coats spotted on filter papers [154]; such methods are greatly recommended nowadays, especially for the international shipment of samples. Many samples can be processed simultaneously, making them potentially suitable for large-scale surveys. However, currently, the cost and complex technology of PCR analyses are still limiting factors for generalized routine use of the test in remote enzootic/endemic areas.

Although DNA detection methods exhibit a higher sensitivity and specificity than parasitological methods, they are affected by the same limitations. Indeed, (i) a negative test cannot ascertain the absence of infection (serological tests are better adapted to do so); and (ii) a positive test ascertains the presence of a specific DNA taxon, but in geographical areas where several pathogenic *Trypanosoma* species co-exist, an animal positive to one taxon may be carrier of one or several others. Users of molecular detection methods must be fully aware of these limitations, which are too often overlooked.

### Antigen detection methods

In addition to detecting either the parasites themselves, or their DNA, antigen detection can also be implemented to evidence active infection. ELISAs for antigen detection based on monoclonal antibodies, which were developed in the 1990s, initially showed promise [155]. However, in field evaluations they were found to present a severe lack of sensitivity and specificity and, hence, were abandoned [6, 156, 157]. Nevertheless, antigen detection methods using monoclonal antibodies would be beneficial and likely to be suitable for the development of rapid tests; if based on cautiously pre-identified trypanosome antigens circulating in the bloodstream (constitutive or secreted), this avenue of research should be encouraged. Such a rapid test could be useful for deciding on treatment, probably alongside HCT and Card agglutination tests, providing they can be developed in other species than *T. evansi*, to complete the panel of ATAO diagnostic tools. For the time being, serological methods for trypanosome are focused on antibody detection.

### Antibody detection methods based on native antigens

Antibody detection methods can provide evidence of a contact between the host and the parasite with very high sensitivity. However, due to the persistence of the antibodies in the blood serum after parasite elimination, such techniques cannot ascertain active infection. They are thus valuable tools in epidemiological surveys and for the detection of suspect animals. They exhibit a high specificity for other genera, such as *Anaplasma*, *Babesia* and *Theileria*, among others, but among the Trypanosomatidae, the specificity is rather low due to patent cross-reactions, as detailed in a subsequent section.

The OIE validated four main antibody detection techniques for routine use: the indirect fluorescent antibody test (IFAT), the whole-cell lysate soluble antigens (WCLSA) antibody-detection ELISA, the complement fixation test (CFT; used for Dourine) and the Card Agglutination Test for Trypanosomes (CATT/*T. evansi* used for Surra). Among these OIE-recommended tests, only the latter test is commercially available (Table 1), making the availability of trypanosome serological diagnostics quite limited. IFAT, CFT and classical ELISAs for the diagnosis of trypanosomosis detect immunoglobulin G (IgG), the levels of which are fairly stable during infection course.

#### Indirect fluorescent antibody tests

The IFATs for trypanosomes exhibit good sensitivity but limited specificity. The main drawbacks of the IFAT is the need for sophisticated microscopy, the subjectivity of the interpretation, which makes the comparison of results quite tricky, and the tiredness of technician’s eyes it generates [158]. Consequently, the performance of the IFAT remains subjective and as such it is not adapted to large-scale studies. The technique is generally used for individual diagnosis, as an alternative to ELISA, and mostly for Dourine.

#### Enzyme-linked immunosorbent assay

The original antibody ELISA for trypanosomoses [159] has been further developed for large-scale surveys in bovines, buffalos, camels, horses and pigs [160, 161]. The standard antigens for ELISA (nowadays called “classical ELISA”) are derived from native trypanosome bloodstream forms produced in laboratory rats and purified by diethylaminoethanol (DEAE) anion-exchange chromatography [99]. They include an extensive range of native antigens that confer the tests a high sensitivity [6, 160, 162, 163]. The classical ELISAs recommended by the OIE have been standardized for camels, cattle, buffalo, elephants, among others [104, 164–166]. The result of an ELISA is considered to be positive when the optical density of the sample is higher than the positive threshold previously defined (Fig. 7c).

Using the ELISA, positive seroconversion occurs generally and, on average, around 10–20 days after infection. After a fully curative treatment, negative seroconversion has been observed within 3–4 months in young and adult animals [167] and after 6 months in older individuals [6, 168, 169], although some authors claim it might take up to 13 months [170]. Based on these data, adequate sampling and proper knowledge of trypanocide use facilitate the correct interpretation of the test results; for example, a serological test implemented 6 months after treatment would confirm treatment efficacy.

Antibody-detection ELISAs have high sensitivity and are better suited than parasitological and PCR techniques to establish the prevalence of infected animals. In addition, their genus specificity is high, meaning that infection with other hemoparasites, such as *Theileria theileri*, *Theileria mutans, Babesia divergens* or *Anaplasma marginale*, do not cause cross-reactions in serological tests against pathogenic trypanosomes [159]; even *Trypanosoma theileri*, the non-pathogenic* Megatrypanum* found in Bovinae, does not cross-react [171, 172]. However, species-specificity among the pathogenic trypanosomes is generally low due to strong cross-reactions between the main parasites *T. vivax*, *Trypanozoon* and *T. congolense* sensu lato (s.l.) [173].

Immunodiagnostics by ELISA requires expertise and relatively expensive and sophisticated equipment, both of which are not always readily available. The technique also involves the production of native parasites to prepare soluble antigens from whole-cell lysate of the trypanosomes, which are not commercially available. In practice, trypanosome antigen production is limited to specialized laboratories. At the individual level in the field, in terms of test implementation there is a substantial delay between the actual sampling and the availability of the results. Moreover, a cut-off value must be defined and the ELISA adapted, evaluated and validated for each host species. All of these factors may present an obstacle to accurate interpretation of the results, given the difficulty of acquiring reference samples for the various species of interest. Nevertheless, the antibody ELISA lends itself to a high degree of automation and standardization that is suitable for sero-epidemiological studies. After collection, serum samples can be stored at − 20 °C or blotted onto filter papers (then stored as dried serum or blood spots) to make the samples more suitable for international shipment. Taken together, the antibody ELISA is an instrumental tool for large-scale surveys to determine the distribution of ATAO (including NTTAT), as well as for post-treatment or post-control campaign follow-ups. Recent work on lyophilized reagents and serum samples has demonstrated that lyophilization is a convenient way to store and ship reagents for ELISAs [174], which should help considerably in further wide-spread implementation of the ELISA technique for trypanosomes in Africa.

Based on in vivo-produced parasites, the classical ELISA is difficult to standardize for high throughput, and this aspect needs improvement. Quality and standardization of the native antigens for ELISAs could be significantly enhanced through the production of the parasite in vitro. WCLSA prepared this way will guarantee a high sensitivity due to the rich panel of native antigens they exhibit and the lower degradation occurring during preparation. This method will allow high standardization and reproducibility of the antigens produced and also solve the ethical problem of using living animals for parasite production.

#### Agglutination tests

Agglutination tests have been developed for trypanosomosis detection; however, most have been abandoned due to poor standardization, with the exception of the CATT for *T. evansi* (CATT/*T. evansi*), which is commercially available from the Institute of Tropical Medicine, Antwerp, Belgium [175, 176]. The antigen of this CATT consists of fixed and stained *T. evansi* Rode *Trypanozoon* antigen type (RoTat) 1.2 parasites produced in rats. The test mainly detects IgMs, which are early-circulating antibodies. IgMs are pentavalent immunoglobulins presenting very high antigen binding affinity; one advantage of these IgMs is that they are prone to form lattices with the antigen. The CATT is carried out on white plastic cards that are rotated for 5 min at 70 revolutions per minute (rpm); the agglutination of stained parasites can be observed by reading with the naked eye (Fig. 7d). A disadvantage of IgMs is that being phagocytized as immune-complexes, their concentration in the serum fluctuates over time, thereby being responsible for false negative test results [177]. As the IgMs have a short half-life, they are a good indicator of a recent infection, or at least of a recent circulation of trypanosomes in the blood. CATT/*T. evansi* is rather inexpensive, fast, simple and can be implemented in the field on any host species. Its sensitivity is generally high in equids, buffalo, camels, sheep, goat and dogs, with a medium specificity. However, once again, although genus specificity is high, species specificity is limited and cross-reactions with other* Trypanozoon* and with *T. vivax* have been reported [41]. Since both specificity and sensitivity of CATT/*T. evansi* are low in cattle and pigs [95, 96], the test is not adapted to large-scale studies of these animals.

A specific CATT/*T. brucei gambiense* is available for diagnostic purposes in humans [178]; however, it has not been largely used in animals [26]. Its level of specificity in animals has not been determined, but it can be presumed to be low because of strong cross-reactions already observed among* Trypanozoon* and between *T. brucei* and other Salivarian trypanosomes [173, 179].

#### Complement fixation test

The CFT allows the detection of *T. equiperdum* (Doflein, 1901) antibodies in both asymptomatic equids and in individuals with clinical signs based on the use of crude antigens derived from the *T. equiperdum* OVI strain ITMAS 241199C that is adapted to rodents [180]. This test has been used to confirm cases of Dourine [181], a disease that is considered by the OIE to be non-treatable [182]. An inter-laboratory ring trial to evaluate CFT for Dourine diagnosis that involved 25 reference laboratories for Dourine confirmed the reliability of this method and the importance of standardizing critical reagents, including the crude antigens and the use of a standard *T. equiperdum* serum across multiple laboratories [92, 183].

#### Trypanolysis test

The trypanolysis test (TL) assesses the presence of specific antibodies through exposure to live *T. evansi* RoTat 1.2 previously grown in mice [184]. The test has been shown to exhibit high specificity, although comparative studies are scarce. As for tsetse-transmitted trypanosomes, other strains, such as LiTat 1.3, 1.5 and 1.6, used for detection of *T. b. gambiense* in humans, can also be used for detection of *T. b. brucei* [29], which tends to show a limited specificity. The TL is rarely used for diagnosis in animals due to it being highly time consuming and costly, with substantial technical constraints and the ethical issue of growing parasites in live animals. Experimental studies are needed to accurately determine the performance of trypanolysis in cattle infected with different *Trypanozoon* [29].

### Antibody detection methods based on purified or recombinant antigens

Several attempts have been made to improve the potential for the standardization of ELISAs through the use of purified or recombinant antigens. Some of these target the VSG, a strategy that could be limited to clonal parasites presenting a highly predominant VAT, such as RoTat 1.2 [184]. The potential limitations of the tests based on VSGs have been recently discussed, with the authors concluding that they cannot be the best tools [33]. Indeed, for *T. evansi,* the ELISA using RoTat 1.2 VSG might be too specific to be able to detect all variants of the taxon, since *T. evansi* type B does not express this gene [185], while it is absent in some other isolates [142]. Surprisingly, a new isolate of *T. equiperdum* was also recently classified as a type B [126]. The reliance on RoTat 1.2 VSG as the basis for diagnosis thus undoubtedly means that non-RoTat 1.2 *T. evansi* will not be detected [119, 143]. Regarding other *Trypanosoma* spp., Auty et al. [33] concluded that “*the overall within-species diversity in both T. congolense and T. vivax [repertoire] probably means that a ‘catch-all’ diagnostic test based on VSGs is unlikely to be successful*”.

Attempts at developing diagnostic tests for *T. evansi* using invariant antigens were recently made. Although the authors claimed the tests were very sensitive and specific, comprehensive evaluation is still needed, and it is therefore too early to draw conclusions on their value [186].

Other attempts at recombinant antigen-based techniques have been made for the development of rapid tests; promising as far as standardization is concerned, they generally present a lower sensitivity, but exhibit a higher specificity when a highly species-specific antigen is selected [86, 87, 187–189]. However, recombinant antigen techniques are generally based on a single molecule harboring a very limited epitope diversity; as such, they can hardly compete with native antigens in terms of sensitivity. Still, such methods could be helpful to develop highly species-specific tests to be used as a second diagnosis step. For example, in the case of *T. cruzi*, such tests could allow the diagnostics after screening to be refined with a more sensitive tool based on cross-reactions with native antigens of *T. evansi* [190]. On the other hand, a recent study has shown that the sensitivity of recombinant antigen-based ELISA could be markedly enhanced by combining several recombinant antigens, which opens the door to further improvements [191].

Other tests have been developed or are under development to target more specific antigens, such as the RDT (rapid diagnosis test) for *T. vivax* and *T. congolense* [87]. Recently, an antigen capture-ELISA test for *T. vi*vax and *T. congolense* using TvGM6 and TcCB1 proteins as antigens, respectively, has been introduced commercially as an RDT (CEVA; Ceva Santé Animale, Libourne, France) [82, 87]. It is the only RDT to reach this stage for the diagnosis of animal African trypanosomosis (AAT), but is still not widely available. In addition, it lacks sensitivity in very early and late infections, and it has the same drawbacks as other antibody-detecting tests in terms of specificity as it will also detect recent other than past infections, which significantly reduces its relevance for treatment decision-making. As the RDT format is primarily relevant for diagnosis at the individual level in the field (pen-side test), it is to be feared that only tests based on antigen detection will be of real commercial interest.

Finally, RDTs for the detection of the human parasites *T. b. gambiense* were evaluated in animals, while the sensitivity was medium to high, specificity was not satisfying, with the authors concluding that “*the SD BIOLINE HAT® is not suitable for screening of T. b. gambiense in domestic livestock*” [192, 193].

The OIE-recommended ELISA based on WCLSA remains the best tool for antibody detection with optimal sensitivity. The improvements required on this technique can now be fulfilled, thanks to the use of lyophilized reagents and dry samples, and to the in vitro production of most of the ATAO. It can then be expected, in the near future, that WCLSA will be prepared from well-standardized in vitro-produced parasites.

### Conclusions on serological diagnosis

Serological methods for trypanosomoses appear useful, thanks to their high sensitivity and specificity regarding the pathogenic trypanosomes (*T. theileri*-infected animals do not show seropositivity). However, cross-reactions are very strong between pathogenic trypanosomes [173], or even *Leishmania*, in humans and animals [69, 194, 195]. Consequently, routine antibody detection methods for trypanosomoses are not species specific. Thus, the seropositivity of an animal to one of the antibody detection methods used (*T. vivax*,* T. evansi*,* T. brucei*,* T. congolense*,* Leishmania*,* T. cruzi*, etc.) must be interpreted as a positivity to one or several of the *Trypanosoma* and/or *Leishmania* species present in the geographical area (see Fig. 5). For example, in the Americas, a dog testing seropositive to *Leishmania* may be—or may have been—infected “either or/and” by *Leishmania*, *T. evansi* and *T. cruzi*. In Africa, when seropositive to *T. vivax* ELISA, bovines can be considered as “are currently, or have been infected”, “either or/and” by *T. vivax*, *Leishmania, T. congolense* s.l., *T. brucei* spp*.* and/or *T. evansi.* Users of serological detection methods should be fully aware of this limitation, which is too often overlooked.

## Tools for the detection of animal trypanosomes in humans

Typical human trypanosomes are *T. b. gambiense* and *T. b. rhodesiense* in Africa, and *T. cruzi* in the Americas. Their specific diagnosis methods, quite similar to those used in animals, is reviewed elsewhere (see “Proper use and perspective on diagnosis methods for animal trypanosomoses” [51]). Briefly, they include: (i) clinical suspicions; (ii) parasitological techniques (direct blood or lymph microscopic examination, HCT and mAECT) [196, 197]; and (iii) serological techniques, such as IFAT, ELISA, CATT/*T. b. gambiense*, RDTs and TL specific for sleeping sickness [198–202]. With the exception of the latter, which exhibits high species specificity, these serological tests have low species specificity. However, in a given epidemiological setting, where only one pathogenic trypanosome species or subspecies is expected in humans, a genus-specific diagnosis is sufficient for population screening. Molecular techniques are also useful in conjunction with mAECT to detect *T. brucei* spp. [203]. Therefore, the use of these tools must be tailored to the final purpose of the investigation, which may be an epidemiological study, blood bank screening or individual diagnosis for treatment decision-making or follow-up.

Although humans have innate immunity against most animal trypanosomes [204], a few cases of human infections with animal trypanosomes have been reported. A small part was due to *T. vivax* and *T. congolense*, but most of the confirmed cases were due to *T. evansi* (agent of the Surra in animals) and *T. lewisi* (parasite of rats transmitted by fleas), which were detected occasionally in Africa, but mostly in Asia [12]. In particular, two *T. evansi* cases of febrile episodes were detected by microscope examination of blood and confirmed by molecular assays in India in 2004 [205, 206] and in Vietnam in 2015 [207]. For the diagnosis of *T. evansi* in humans, CATT/*T. evansi*, ELISA *T. evansi*, parasitological techniques and PCR commonly used for animals were applied (with only slight modifications for ELISA) [207].

For *T. lewisi*, a dozen cases have been described with variable issues: self-cure in some cases, successful treatment using drugs effective for HAT (pentamidine, suramine, melarsoprol) or death [208]. However, recent observations demonstrated that none of these trypanocides was efficient against a *T. lewisi* isolated in Thailand [209]. Moreover, resistance testing to normal human serum reveals that *T. lewisi* is a potentially underestimated human pathogen [210]. The increase of anthropization and the presence of invasive rodents dwelling with humans in poor living conditions reinforces the hypothesis of in-door contamination of humans by *T. lewisi* [211].

Pertaining to the diagnosis of *T. lewisi* in humans, most of the known cases have so far been identified by direct microscope observation (Fig. 6) and by molecular techniques; specific primers hybridizing inside the ITS1 were published to detect *T. lewisi* DNA [96]. In Thailand, a 45-day-old infant infected with a *T. lewisi*-like was indeed diagnosed based on the ITS1 sequence [212]. Attempts to develop ELISA *T. lewisi* were recently made with promising results [213]; however, human reference serum samples are still lacking for the standardization of such tests.

## Conclusions

The diagnosis of trypanosomoses can proceed based on evidence of: (i) the parasite itself, with limited sensitivity and specificity; (ii) its DNA, with higher sensitivity and potentially high specificity (although limited within the* Trypanozoon*); (iii) immunoglobulins directed against more or less specific parasite antigens. The characteristics of these tests must be carefully considered for effective application according to the different epidemiological situations. In addition, a comprehensive analysis of the proper use of these diagnostic techniques will be necessary to adapt recommendations and to support the prospects of developing complementary diagnostic methods, including rapid tests that could be applied individually for decision making in the field.

## Data Availability

All data generated or analyzed during this study are included in this published article.

## Abbreviations

AAT: Animal African trypanosomosis
a-HT: Atypical human infections by animal trypanosomes
ATAO: Animal trypanosomoses of African origin
BCM: Buffy coat method
CATT: Card agglutination test
CFT: Complement fixation test
DEAE: Diethylaminoethanol
ELISA: Enzyme-linked immunosorbent assay
FAO: Food and Agriculture Organization of the United Nations
GSBS: Giemsa-stained thin blood smear
HAT: Human African trypanosomiasis
HTC: Hematocrit concentrations technique
IFAT: Indirect fluorescent antibody test
IgG: Immunoglobulin G
IgM: Immunoglobulin M
ITS1: Internal transcribed spacer 1
LAMP: Loop-mediated isothermal amplification
mAECT: Mini anion exchange centrifugation technique
NTD: Neglected tropical disease
NTTAT: Non-tsetse transmitted animal trypanosomosis
PCV: Packed cell volume
OIE: World Organization for Animal Health
RoTat: Rode *Trypanozoon* antigen type
rpm: Revolutions per minute
RT-PCR: Real-time PCR
SL-RNA: Spliced-leader trypanosome RNA
TL: Trypanolysis test
VAT: Variable antigen type
VSG: Variable surface glycoprotein
WCLSA: Whole-cell lysate-soluble antigens
